# Highly Accurate Identification of Bacteria’s Antibiotic Resistance Based on Raman Spectroscopy and U-Net Deep Learning Algorithms

**DOI:** 10.1021/acsomega.2c03856

**Published:** 2022-08-12

**Authors:** Zakarya Al-Shaebi, Fatma Uysal Ciloglu, Mohammed Nasser, Omer Aydin

**Affiliations:** † Department of Biomedical Engineering, Erciyes University, 38039 Kayseri, Turkey; ‡ NanoThera Lab, Drug Application and Research Center (ERFARMA), Erciyes University, 38039 Kayseri, Turkey; § Department of Geomatics Engineering, Erciyes University, 38039 Kayseri, Turkey; ∥ Clinical Engineering Research and Implementation Center, (ERKAM), Erciyes University, 38030 Kayseri, Turkey; ⊥ Nanotechnology Research and Application Center (ERNAM), Erciyes University, 38039 Kayseri, Turkey

## Abstract

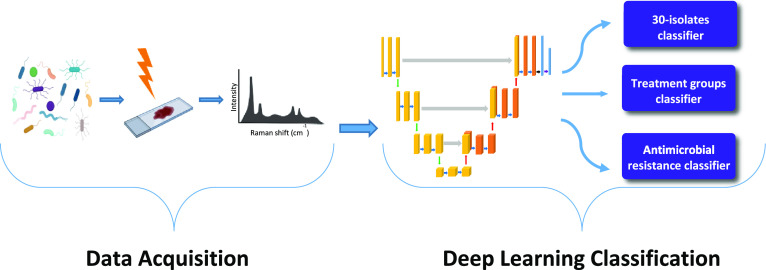

Bacterial pathogens especially antibiotic-resistant ones are a public health concern worldwide. To oppose the morbidity and mortality associated with them, it is critical to select an appropriate antibiotic by performing a rapid bacterial diagnosis. Using a combination of Raman spectroscopy and deep learning algorithms to identify bacteria is a rapid and reliable method. Nevertheless, due to the loss of information during training a model, some deep learning algorithms suffer from low accuracy. Herein, we modify the U-Net architecture to fit our purpose of classifying the one-dimensional Raman spectra. The proposed U-Net model provides highly accurate identification of the 30 isolates of bacteria and yeast, empiric treatment groups, and antimicrobial resistance, thanks to its capability to concatenate and copy important features from the encoder layers to the decoder layers, thereby decreasing the data loss. The accuracies of the model for the 30-isolate level, empiric treatment level, and antimicrobial resistance level tasks are 86.3, 97.84, and 95%, respectively. The proposed deep learning model has a high potential for not only bacterial identification but also for other diagnostic purposes in the biomedical field.

## Introduction

Antibiotic resistance which is a subset of the term antimicrobial resistance (AMR) poses a growing threat to public health around the world and puts a substantial financial strain on global healthcare systems.^1^ Among the top 10 global health threats listed by the World Health Organization (WHO) in 2019, AMR took the fifth place on the list.^2^ Furthermore, in the United States, at least 2.8 million people contract an antibiotic-resistant infection each year, with over 35,000 deaths.^3^ Subsequently, to knock AMR down, it is very important to increase the awareness among people about the peril of misuse and overuse of antibiotic drugs. In addition, it is important to diagnose the infection correctly in order to identify the type of bacteria that causes the infection, thereby using an appropriate antibiotic drug.

Many techniques are used to identify bacteria and detect antibiotic resistance. Although the culture-based approach which is considered the gold standard for antibiotic susceptibility of bacteria is preferred mostly, it requires long incubation times.^4^ Therefore, different techniques are employed to overcome these drawbacks such as mass spectroscopy,^5,6^ enzyme-linked immunosorbent assay (ELISA),^7,8^ and polymer chain reaction (PCR).^9,10^ However, these techniques need complex sample preparation procedures. In addition, they necessitate the use of expensive reagents and instruments and must be operated by trained technicians, raising the entire cost of the analysis.^11^ Hence, it is highly important to find a rapid, easy, and reliable technique to detect bacteria and antibiotic resistance/susceptibility.

Raman spectroscopy is a non-destructive technique and one of the most promising technologies in medical diagnosis. It is based on the interaction of light and molecules within a sample. Therefore, it can provide worthwhile information about the sample under observation by detecting the vibrational modes of the molecules. On the lines of bacterial and antibiotic-resistance detection, Raman spectroscopy has the unique potential to be a technique for identifying phenotypes that do not require specially designed labels,^12,13^ allowing for easy generalizability to new strains. On the other hand, Raman spectroscopy provides weak signal intensity, and the high level of fluorescence leads to noisy spectra that are hard for the discrimination of similar spectral data.^14^ Furthermore, it is too hard to observe the difference in Raman spectra between various species with the naked eye because their compositions are similar.^15^ For example, there are huge similarities in Raman spectra of antibiotic-resistant and susceptible bacteria due to the high biomolecular similarities in these groups. To overcome this limitation, the use of powerful machine learning techniques is indispensable.

As a tool of multivariate modeling processes, traditional machine learning techniques such as the support vector machine (SVM), *k* nearest-neighbor classification model (KNN), naïve Bayes (NB), and random forest (RF) are used to classify spectral data.^16−18^ Stöckel et al.^19^ have used single-cell Raman spectroscopy combined with SVM in order to identify 26 different species of *Mycobacteria tuberculosis*. In the same manner, Yan et al.^20^ have utilized kernel principal component analysis with the decision tree to discriminate 23 common strains of food-borne bacteria. Both studies have demonstrated the ability of traditional machine learning in the identification of microorganisms. Nevertheless, it is well known that deep learning models show superior performance compared to traditional machine learning algorithms on large data sets.

Ciloglu et al.^21^ have used stacked-autoencoder (SAE)-based deep neural networks to classify antibiotic-resistant and susceptible strains of *Staphylococcus aureus* (*S. aureus*) bacteria. The authors have demonstrated that the deep learning model is more accurate than the traditional machine learning classifiers. Tang et al.^22^ have compared the traditional machine learning and deep learning algorithms for the classification of nine *Staphylococcus* species, and the results of this study support the fact of that deep learning is superior to traditional machine learning. Moreover, there are some studies in the literature that used convolutional neural network (CNN) architecture for one-dimensional (1D) spectral classification, especially for Raman spectra.^12,23−27^ Lu et al.^27^ have presented a new approach for identifying spectra of 14 microbes by using laser tweezers Raman spectroscopy combined with a CNN, and they demonstrated the ability of CNNs to classify the different types of microbes. Recently, Ho et al.^12^ have collected large data from 30 different classes of microorganisms using Raman spectroscopy (from now on, it will be referred to as the Stanford Data). Using the residual neural network (ResNet) with Raman spectroscopy revealed a striking ability to distinguish 30 classes with fair accuracy. In addition, Deng et al.^25^ have used Stanford Data with a different deep learning model. The study has established a multi-scale 1D CNN model in order to collect more information on the Raman spectra and thereby improve the accuracy of bacterial identification. The study, indeed, has got good results compared to those of the ResNet model. Nevertheless, the model could not classify some important classes very well such as methicillin-resistant *S. aureus* (MRSA), methicillin-sensitive *S. aureus* (MSSA), *Escherichia coli* (*E. coli*), and *Klebsiella pneumoniae* (*K. pneumoniae*).

In deep learning models, when the layer is deep, neural networks have a disadvantage in that they do not learn well. To overcome this flaw, ResNet wages a skip connection, in which the input is appended to the same layer’s output.^28^ This approach enables the gradients to flow through a network that directly feeds the output of one layer as the input to the next layers, which aids learning in more complex architectures.^29^

As an improvement and development of fully convolutional networks (FCNs), U-Net architecture was designed for medical image segmentation by Ronneberger et al.^30^ The architecture mainly depends on the encoder–decoder technique with the aid of skip connections, which provides high flexibility and performance.^31^ The fundamental difference between the U-Net architecture and other FCNs is the addition of successive layers to a traditional contracting network, where pooling operations are substituted with up-sampling operators. As a result, depending on this knowledge, a subsequent convolutional layer can learn to create an accurate output.^30^ Additionally, as it is needed in segmentation, U-Net has low-level detailed information and high-level feature maps, making it achieve high performance in classification tasks.^32^ Moreover, U-Net can be trained end-to-end with a small data set, and the input and output dimensions of the network are kept consistent with the architecture. Gebrekidan et al.^33^ have used U-Net architecture to refine Raman spectra. They presented an automated U-Net-based approach for noise and background removal or reduction, and the U-Net architecture demonstrated its abilities to deal with Raman spectra and refine them.

In this study, the U-Net architecture was constructed based on neural network backbones to reveal the bacterial Raman fingerprint information, which is made up of spectral peaks of varied wavenumbers and widths, and then properly classify them. The large Stanford Data^12^ were used in this study, which include 30 common bacterial pathogens. Data augmentation was utilized to increase the number of the bacterial data set and improve the diversity of the data set by adding noise to the original Raman spectra. The proposed model’s main goal is to classify Raman spectra of bacterial antibiotic resistance, which is highly important to be identified correctly to knock the pandemic of antibiotic resistance down. Besides the antibiotic resistance classification, the deep learning model was exploited to classify two tasks: classification of 30 isolates of bacteria and empiric group classification. To the best of our knowledge, this is the first study that used U-Net architecture to classify Raman spectra. The findings of this study show that Raman spectroscopy combined with U-Net architecture provides successful results for the bacterial species classification in different tasks, especially in antibiotic resistance and susceptibility classification.

## Results and Discussion

### Performance and Stability of the Model

**Figure 1:**
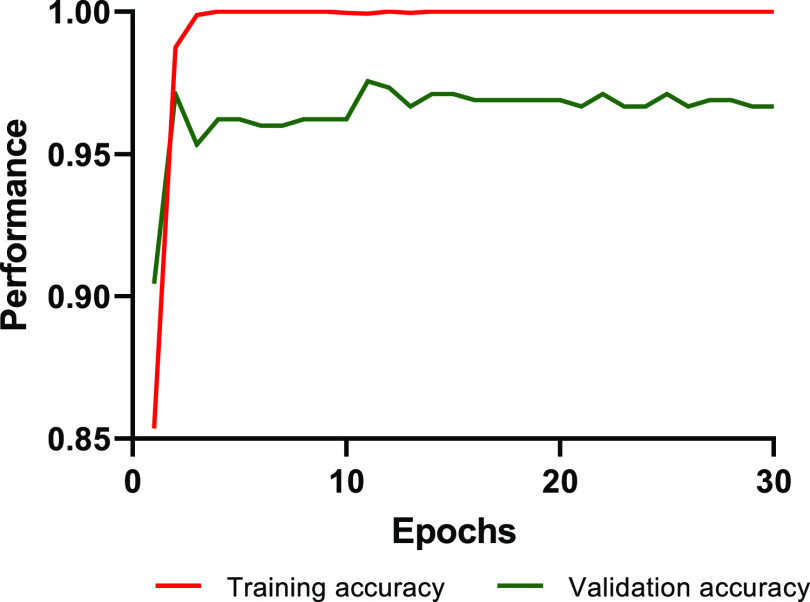
Plot of the training accuracy and validation accuracy.

This study used U-Net architecture to extract significant features in Raman spectra of bacteria in order to make the classification process more accurate. U-Net architecture can solve problems that some deep learning algorithms suffer from. For example, data could be missed when the deep learning layers are so deep. Therefore, some architectures have been performed to solve this shortcoming by summing the output and input in each layer. However, they faced another problem that the first layers became weaker. To illustrate the U-Net model training process stability, the learning curve of the fine-tuning phase of the 30-isolate classifier has been plotted in Figure 1. The model’s performance improved with epochs, implying that it became better with training. It also can be seen that it rose and then became steady, indicating that it was no longer able to learn and reach the convergence state. Moreover, since the training and validation accuracy are both high regardless of the first epochs and they remain nearly constant, the model is not over-fitting because over-fitting can be spotted when the training accuracy and validation accuracy start diverging.

### Identification of Bacterial Isolates and Empiric Treatments

**Figure 2:**
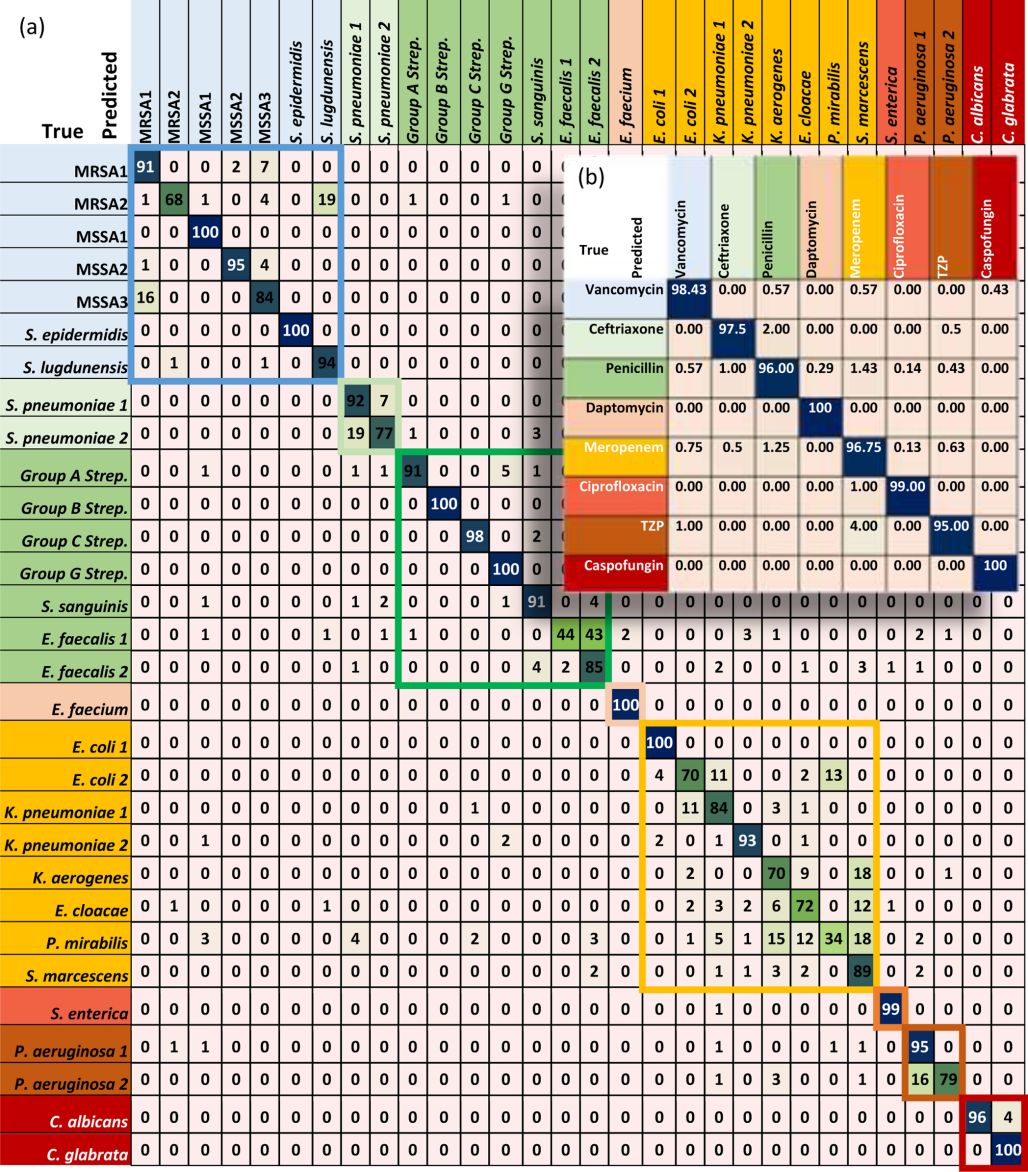
Confusion matrices of the U-Net model for (a) 30 isolates and (b) Empiric treatment. The eight-color boxes are the treatment groups in which every box contains one or multiple strains that were treated with the same drug. (TZP) refers to piperacillin–tazobactam.

Thanks to its structure that enables it to concatenate the feature maps of all preceding layers, the U-Net model has achieved good results. The accuracy is 86.3% in the 30-isolate classifier after testing the model with 3000 spectra. Table 1 shows a comparison of 30-isolates and empiric treatment accuracies in the ResNet,^12^ multi-scale,^25^ and U-Net model. The confusion matrix of the U-Net model for the 30 isolates is shown in Figure 2a. The test data for each class (which are 100 spectra) were distributed very well through the grouping treatment. However, from the confusion matrix (Figure 2a), misclassifications can be seen within a treatment group. For instance, the first treatment group which includes the most common antibiotic resistance and susceptibility (MRSA and MSSA isolates) shows an irregular distribution of the data. Nevertheless, this disorder of the distribution is less than that in ResNet and multi-scale models according to their confusion matrices.^12,25^

**Table 1:** Comparison of the Performance (Accuracy) of the Three Models That Used the Same Data

model	30-isolates classifier (%)	empiric treatment classifier (%)
U-Net	86.3	97.84
multi-scale	86.7	98.0
ResNet	84.2	97.6

It can be observed that the interference between the MRSA and MSSA classes decreased in the U-Net model. In addition, although the Meropenem group has the worst misclassification among its classes, the U-Net model has a lower misclassification compared with the ResNet and multi-scale models inside this class, especially in the Gram-negative *E. coli*, which because of its antibiotic resistance forms serious perils in both human health and hospitals, making it a priority to be identified.^34,35^ However, the multi-scale model has classified penicillin and TZP groups better than the U-Net model. In general, the U-Net model has demonstrated its ability to classify classes within a treatment group just as the multi-scale model does.

In the U-Net model classification of the antibiotic treatment groups, the accuracy is found to be 97.84%, which shows the ability of the model to provide the correct recommended antibiotic treatment. The confusion matrix of the eight treatment groups is shown in Figure 2b. Based on the confusion matrices for this study and other compared models,^12,25^ it can be seen that there is no significant difference among them, which indicates the superiority of deep learning in this task.

### Identification of the Antibiotic Resistance and Susceptibility (MRSA and MSSA)

Antibiotic resistance and susceptibility identification, in addition to empiric treatment, is another resource for fathoming more about bacteria and determining which antibiotic will suppress the growth of the bacteria causing a certain infection. Moreover, according to many health care organizations such as the Centers for Disease Control and Prevention (CDC), antibiotic resistance including MRSA is responsible for causing severe health problems,^36^ and the misdiagnosis between them and antibiotic susceptibility, such as MSSA, leads to serious plight as well. Therefore, there is an urgent need to classify MRSA and MSSA, which are well-known antibiotic-resistant/susceptible bacteria. The Stanford Data include three isolates of MRSA and two isolates of MSSA. These two types of antibiotic resistance and susceptibility data, besides the augmentation data, were used to train, fine-tuning, and test the binary classifier with the same procedures as the 30-isolate classifier. Sufficient antibiotic resistance and susceptibility data were obtained from three different measurement times and five isolates in order to make the results more feasible for generalization.

**Figure 3:**
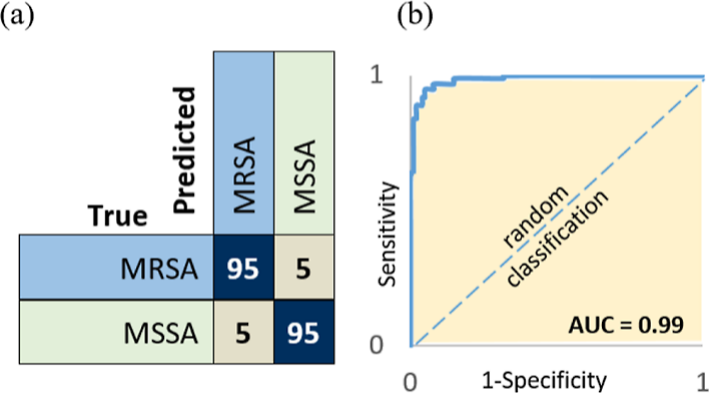
Binary classifier results of MRSA and MSSA. (a) Confusion matrix and (b) Receiver operating characteristic (ROC) curve.

The accuracy of the binary classifier of the U-Net was up to 95%, which exceeds the accuracies of the ResNet and multi-scale as shown in Table 2. Figure 3a illustrates the classification of the MRSA and MSSA within the confusion matrix. It can be seen that the data were distributed equally in the confusion matrix, which reduces the likelihood of misclassification in each class.

**Table 2:** Comparison of the Binary Classifier Performance of the Three Models That Used the Same Data

model	accuracy (%)	AUC
U-Net	95	0.99
multi-scale	92.9	0.98
ResNet	90.4	0.974

Besides the accuracy, the ROC curve, which is a graphical representation of a binary classifier system’s diagnostic capacity when its discriminating threshold is changed, was used to test the performance of the classifier. As shown in Figure 3b, the ROC curve shows the trade-off between sensitivity (true positive rate) and specificity (1 – false positive rate), and the curve was close to the top-left corner, indicating a better performance of the classifier. Additionally, the area under curve (AUC) value is 0.99. In this task, among the three compared models, U-Net has achieved the highest accuracy and AUC value, which makes it the candidate for clinical diagnosis and treatment for bacterial infections.

This is an important outcome since the detection of bacteria’s antibiotic resistance with high accuracy provides correct drug selection and eventually slows down the pandemic of antibiotic resistance.

In clinical practice, users may only be interested in the correct predictions among the samples of a model in the measurements. However, only one value is not particularly enough to trust models’ performances. Therefore, some statistical measurements should be taken into consideration to make a deep learning model reliable. The accuracy, which tests a model’s ability to predict all correct samples whether they are positive or negative, is the most common performance evaluation criterion. In this study, the accuracy of the binary classifier is obtained according to eq 1. Besides accuracy, some statistical analyses were carried out such as recall, precision, Jaccard index, and *F*_1_-score according to the confusion matrix.

The recall or sensitivity of a model (eq 2) measures the capability of the model to predict correct positive samples, while the precision or the positive predictive value (eq 3) measures the true positive values among all the positive samples. Nevertheless, in practice, it is difficult to compare two models using recall and precision because there is a trade-off between them; when the recall increases, the precision decreases and vice versa. Therefore, the *F*_1_-score is used to compare two models regarding recall and precision values. The higher the *F*_1_-score, the better the performance. The *F*_1_-score can be calculated using eq 4. Moreover, the Jaccard index measures the true positive samples among all positive samples, either true positive samples or predictive positive samples. The Jaccard index can be calculated using eq 5. Table 3 shows the results of the abovementioned measurements for the three deep learning models in the antibiotic resistance task.
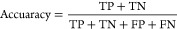
1
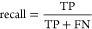
2
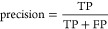
3
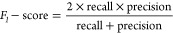
4
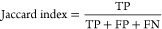
5where TP is the true positive values, TN is the true negative values, FP is the false positive values, and FN is the false negative values.

**Table 3:** Statistical Measurements for all Compared Models

model	recall (%)	precision (%)	*F*_1_-score (%)	Jaccard index (%)
U-Net	95	95	95	90.48
multi-scale	88.62	97.04	92.77	86.52
ResNet	87.38	90	88.67	79.65

### Data Augmentation Impact

The use of data augmentation during training reduces the chance of over-fitting and increases the generalizability of models.^25,37^ The fundamental limitation of data augmentation is that the data derived from the noise added to the original data, that is, the augmented data distribution can deviate significantly from the original. The model may then mistakenly identify the augmentation data instead of the original data, resulting in misclassification and low reproducibility. To get over this limitation, only the fine-tuning data that needed to be extended were augmented.

After augmentation, U-Net performance has increased remarkably. In the binary classifier, the accuracy increased to 95%, whereas the accuracies of the 30 isolates and empiric treatment slightly improved to 86.3 and 97.84%, respectively, as can be seen in Table 4.

**Table 4:** Performance Comparison before and after Using Data Augmentation

model	data augmentation	30-isolates classifier (%)	empiric treatment (%)	binary classifier (%)
U-Net	before	85.13	97.7	93
	after	86.3	97.84	95

Based on all the results obtained in this study, Raman spectroscopy combined with deep learning proves to be a rapid and reliable method to detect and identify bacterial species, which was proved by some studies,^13,38^ including antibiotic resistant/susceptible, and it provides more rapid results than the culture-based method that is used as the gold standard for bacterial identification. Furthermore, Raman spectroscopy allows easy generalizability to new strains compared to other methods since it does not require specially designed labels. All in all, it is believed that the proposed method is a promising tool to identify bacteria and it can be extended to clinical use.

## Conclusions

As bacterial infection including antibiotic resistance has become a serious problem that should be knocked out, the non-destructive Raman spectroscopy is the proposed technique to tackle this problem. However, Raman spectroscopy produces sets of spectra with subtle differences that are difficult to distinguish. Therefore, deep learning, which is a dependable tool to extract the main differences among the Raman spectra, has been used to classify the Raman spectra of different classes. Herein, U-Net architecture has been used and re-engineered to suit 1D data classification. The model has been trained and tested on the Stanford Data, and its performance was pretty well in the three tasks (30-isolate classifier, antibiotic susceptibility binary classifier, and empiric treatment). The accuracies for the three tasks are 86.3, 95, and 97.84% for the 30-isolate classifier, antibiotic susceptibility binary classifier, and empiric treatment, respectively. Although the multi-scale model has slightly better accuracies than the U-Net model in the 30-isolate classifier and empiric treatment, the misclassification within an empiric treatment group is less in the U-Net architecture in some important groups. Finally, U-Net architecture is thought to have a wide range of applications in the biomedical field, including not only the detection of bacteria but also a wide variety of Raman spectroscopy applications.

## Methods

### Data Set

The CNN needs massive data sets to show high performance in classification. In this study, the Stanford Data for 30 classes of bacteria and yeast, which are reflective of the majority of infections in intensive care units around the world,^12^ have been used. The Stanford Data have been collected from three measurement times, and they include information about the isolate level, species level, and antibiotic susceptibility level. In the data (shown in Table 5), there are 60,000 Raman spectra (reference record) for the 30 bacterial classes including MRSA and MSSA, which are commonly antibiotic-resistant and associated with several difficult-to-treat infections.^39,40^ Each class of the 30 isolates contains 2000 spectra in the reference record, which is a satisfying number for training in deep learning. Besides the reference record, there are also 3000 records for fine-tuning, which is the second training phase. In addition, there are 3000 test records for all classes in order to evaluate the model.

**Table 5:** Stanford Data That are Used in This Study

dataset	number of spectra
reference record	60,000
fine-tuning record	3000
tests record	3000

It is worth noting that the 30 isolates were empirically treated with eight different antibiotics (vancomycin, ceftriaxone, penicillin, daptomycin, meropenem, ciprofloxacin, TZP, and caspofungin). Therefore, based on the indicated empirical treatment, the 30 bacteria isolates can be divided into eight groups.

All spectra were chosen to have a wavenumber range of around 400–1800 cm^–1^, which is considered a useful range for studying practically all microorganisms.^41,42^ Moreover, because the optical systems’ efficiency was deteriorating, the measurement period was lengthened to verify that the SNR was consistent between subsets. Furthermore, Raman spectra were preprocessed individually using a polynomial fit of order 5 to correct the baselines of the spectra and normalize the spectra in values between zero and one. Also, 25 spectra with the highest intensity were excluded.^12^

### Model Architecture

The U-Net architecture is a fast and precise CNN that is generally used for 2D medical image segmentation. The model in this study follows U-Net architecture but in one dimension. Every 2D layer in the standard U-Net segmentation model is replaced with its corresponding in 1D space. For the purpose of classification, some modifications were carried out to the architecture such as adding two dense layers and a final classification layer and using the softmax activation function.

**Figure 4:**
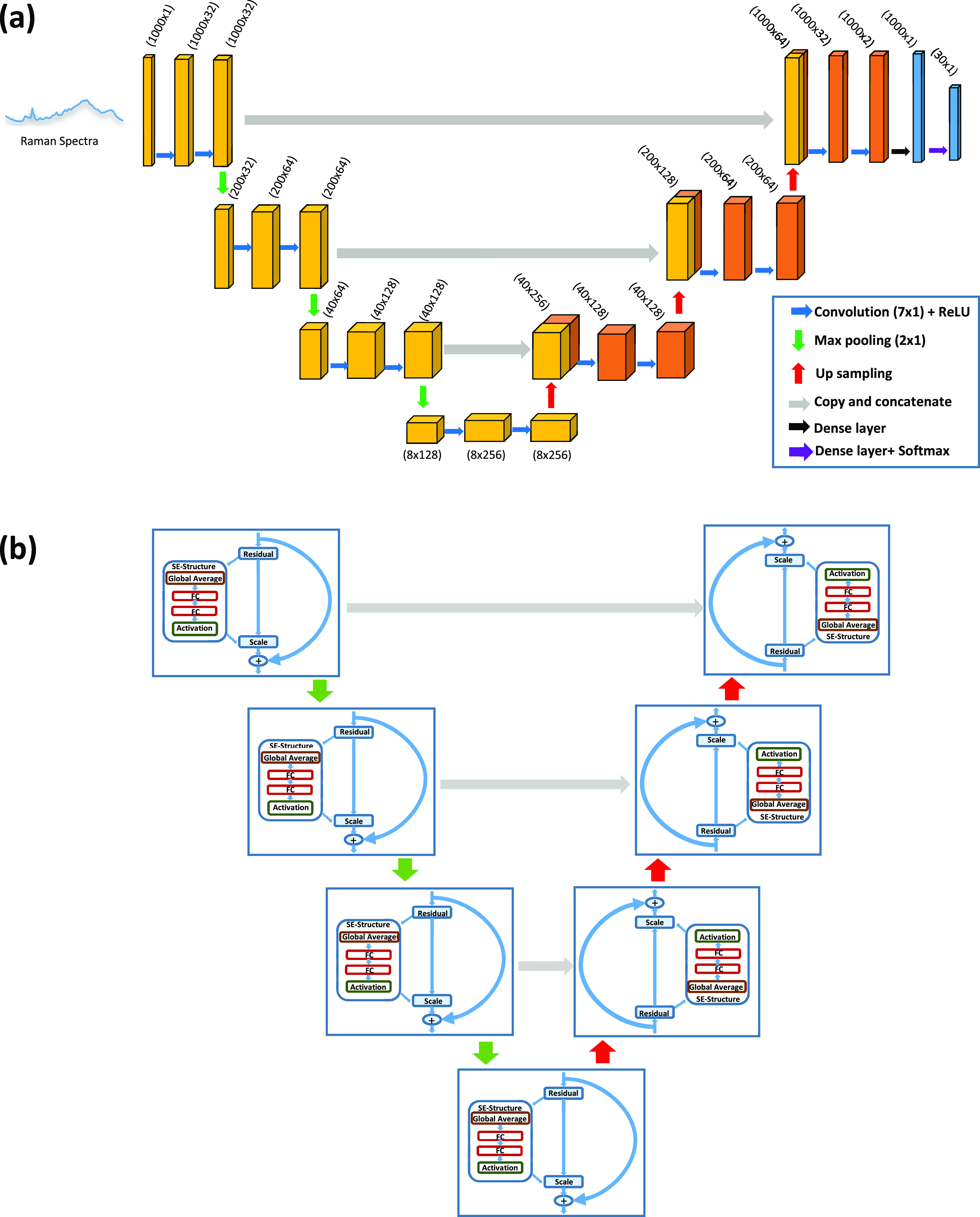
(a) Architecture of 1-D U-Net. The 1D spectra are introduced to the encoder part on the left side of the architecture. As the spectra go down, the number of filters increases with the convolution layers (blue arrows) and the data decrease with the max poling (green arrows). When the data reach the bottom, the decoder with up-sampling (red arrows) increases the data again. The gray arrows represent the copy and concatenation task. Then, in the end, there is a dense layer followed by a dense layer with a softmax (black and purple arrows) for the purpose of classification. (b) General schematic chart of the data influx by the U-Net combined with neural networks and backbones (SE-ResNet).

The U-Net architecture consists of two main components (encoder and decoder), which make it a U-shaped architecture as shown in Figure 4a. In the encoder (the left part of the architecture), the down-sampling reduces input complexity and spatial information in order to increase the feature extraction by doubling the number of filters in every layer, starting with 32 filters in the first convolution layer. The convolutional layers with kernel size 7 extract progressively abstract representations of the input data over numerous steps. In addition, every convolution layer (the blue arrows) in the encoder is followed by a rectified linear activation function (ReLU) and max pooling operations (the green arrows) with two stride intervals. On the other side (the right part of the architecture), the decoder increases the feature and spatial information via a set of up-sampling layers (the red arrows) followed by concatenations (the gray arrows) with high-resolution features from the encoder and convolutions layers with a kernel size of 7. Two convolution layers are applied after each concatenation followed by an activation function (ReLU). Each 32-component feature vector is mapped to the desired number of classes using the convolution operation with a kernel size of 7 at the final layer.

To augment and improve feature extraction from Raman spectra, the U-Net architecture is combined with squeeze-and-excitation residual networks (SE-ResNet) as backbones Figure 4b. Improved channel interdependencies are made possible by using squeeze-and-excitation networks (SENets), a CNN building piece that uses essentially no computational resources. They are simple to integrate into current designs and provide a significant performance gain. A squeezing layer in the SENet block uses average pooling to reduce each channel to a single numeric value. The required nonlinearity is then added using a ReLU function, which is followed by two fully connected layers. Each channel’s gating action is smoothed down by a sigmoid activation at the end. Each ResNet block has two connections from its input, with one connection skipping through the convolutions and functions and the other connection passing through a succession of batch normalization, linear functions, and convolutions. Identity, cross, and skip connections are the names given to these processes. The combined tensor outputs of the two connections are added together.

For classification, a dense layer is added at the end of the model, followed by a dense layer with a softmax activation function.

In the convolutional layers, the trainable convolution operation is undergone as it is illustrated in eq 7
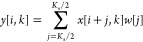
6where *y* is the output of the convolution, *x*is the output of the previous layer, *w*is the trainable convolutional filter, *k* ∈ [0,*K*] is the current feature map, *i* ∈ [0,*N*] is the current element of *x*, and *K*_s_ is the kernel size.^43^

Afterward, the output of the convolution is next submitted to a non-linear activation function *H* (ReLU)
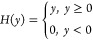
7

As a result, the purpose of the training technique is to learn a set of weights *w* for each convolutional layer for the combined operations to produce the required outputs.

### Training and Evaluation of the Model

The data set had already undergone some preprocessing such as normalization and a polynomial fitting to improve the model performance and avoid over-fitting. In the 30-isolate classifier, 60,000 spectra of reference records were used to train the model as the first training in 30 epochs. In this step, the model learned to extract the features. Then, the second training (fine-tuning) with 3000 spectra was carried out for the model’s parameters in order to be modified very precisely to suit certain observations. However, due to the small size of fine-tuning data, data augmentation was used to increase the data and increase the generalizability of the training model. Augmentation was applied for 50% of fine-tuning data by adding Gaussian noise with a mean of 0.0 and a standard deviation of less than 0.03. In addition, 10% of the fine-tuning data (including the augmentation data) was taken to verify the model performance. The fine-tuning phase was performed in also 30 epochs. The model was able to save the best weights by capturing the best validation accuracy in a certain epoch during the 30 epochs. After the model was trained on the reference and fine-tuning records, it was tested on independent test data acquired from separately cultured samples, and the same data were used to construct the confusion matrix, which is an excellent indicator for classification performance. The same steps were carried out on the binary classifier of MRSA and MSSA. Nevertheless, because the number of fine-tuning data of the binary classifier was very small, augmentation was applied for 80% of the data. All classification procedures were carried out with Python language using TensorFlow^44^ and Keras.^45^ The Adam optimizer^46^ was used in training phases. The learning rate in all phases and classifiers is the same (0.0001).

## Abbreviations

AMR: antimicrobial resistance
WHO: World Health Organization
ELISA: enzyme-linked immunosorbent assay
PCR: polymer chain reaction
SNR: signal-to-noise ratio
SVM: support vector machine
kNN: *k* nearest-neighbor
NB: naïve Bayes
RF: random forest
SAE: stacked-autoencoder
CNN: convolutional neural network
1D: one-dimensional
FCNs: fully convolutional networks
MRSA: methicillin-resistant *S. Aureus*
MSSA: methicillin-sensitive *S. Aureus*
ReLU: rectified linear activation function
*E. coli*: *Escherichia Coli*
ROC: receiver operating characteristic
*Staphylococcus aureus*: *S. aureus*
*K.pneumoniae*: *Klebsiella pneumoniae*
CDC: Centers for Disease Control and Prevention
